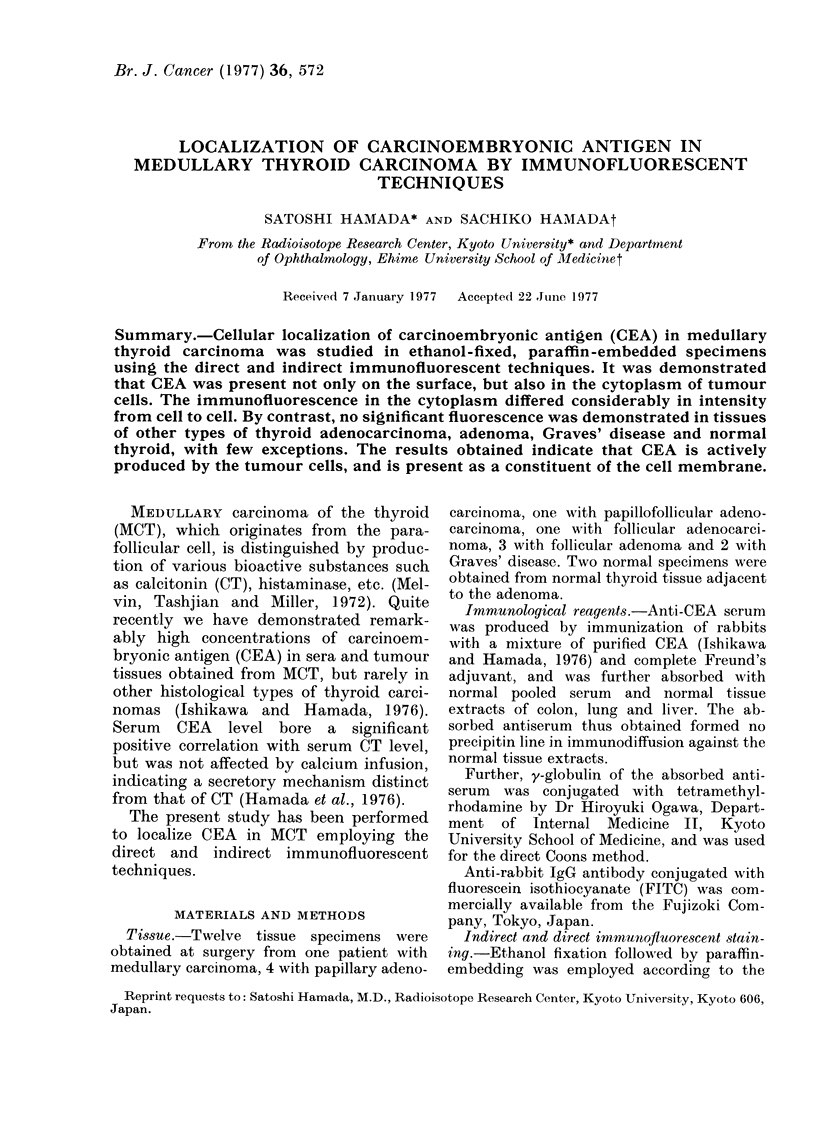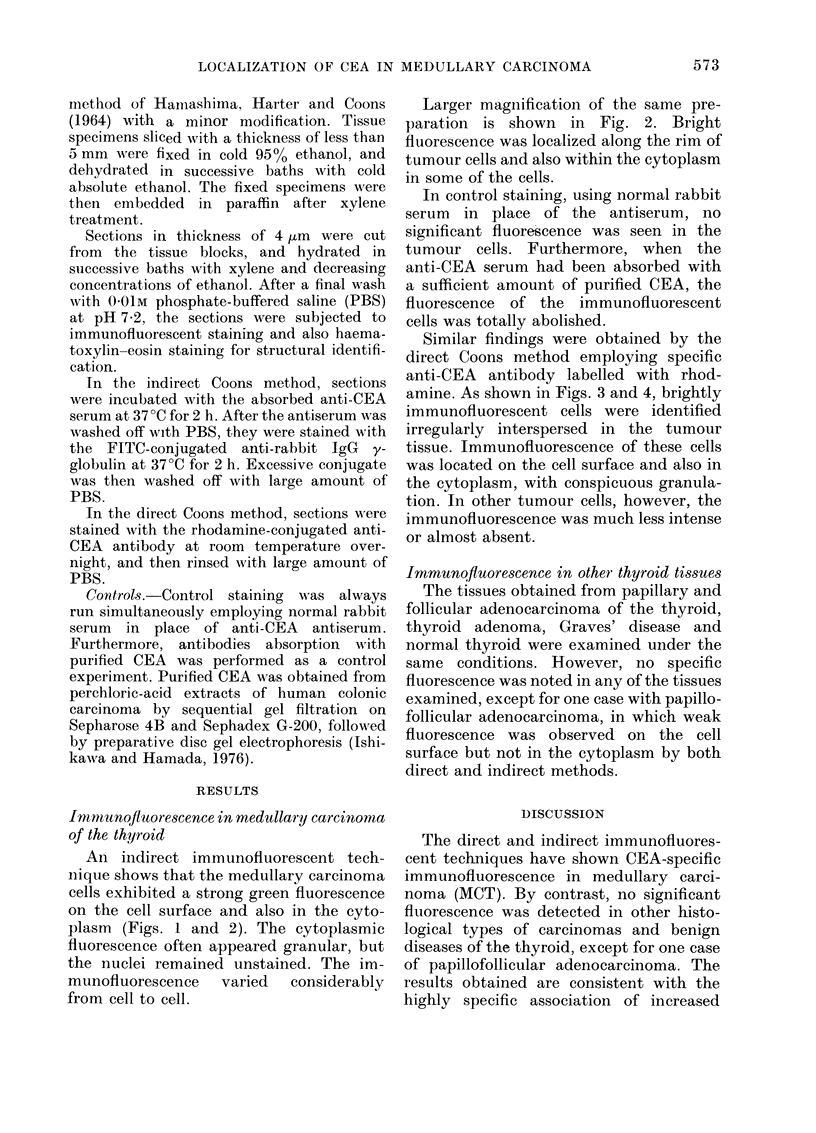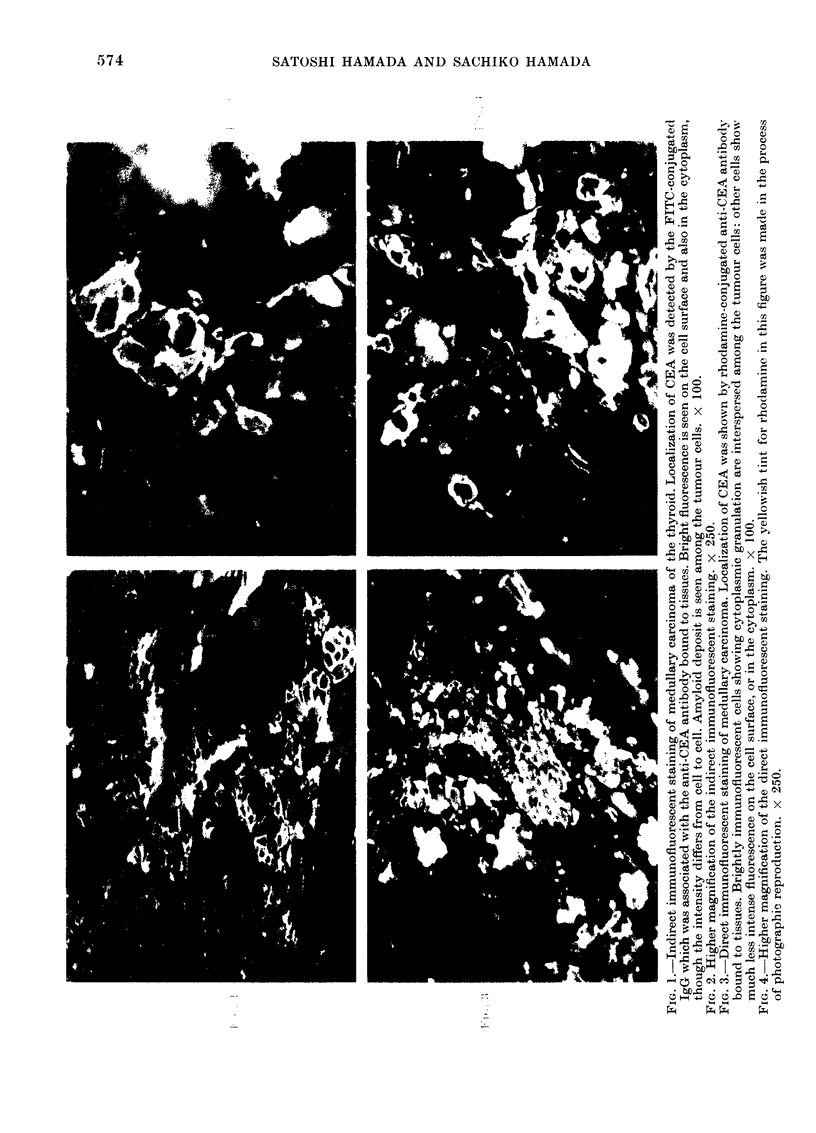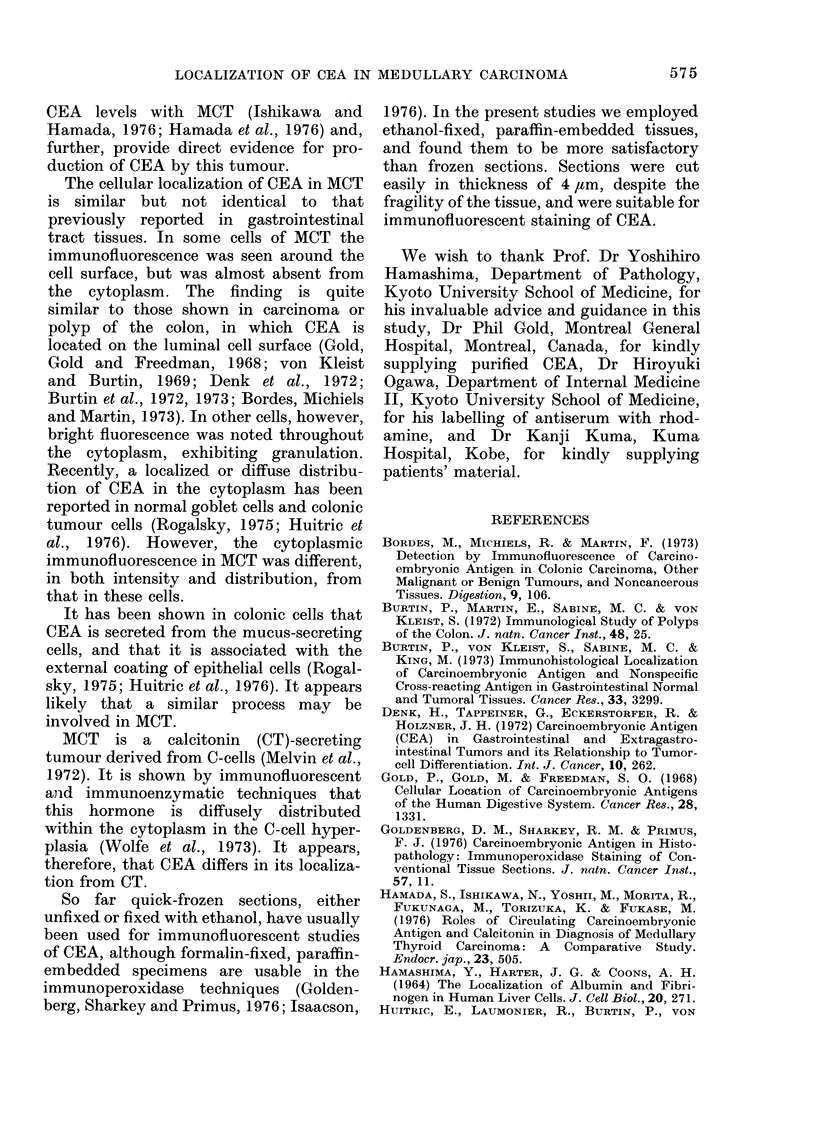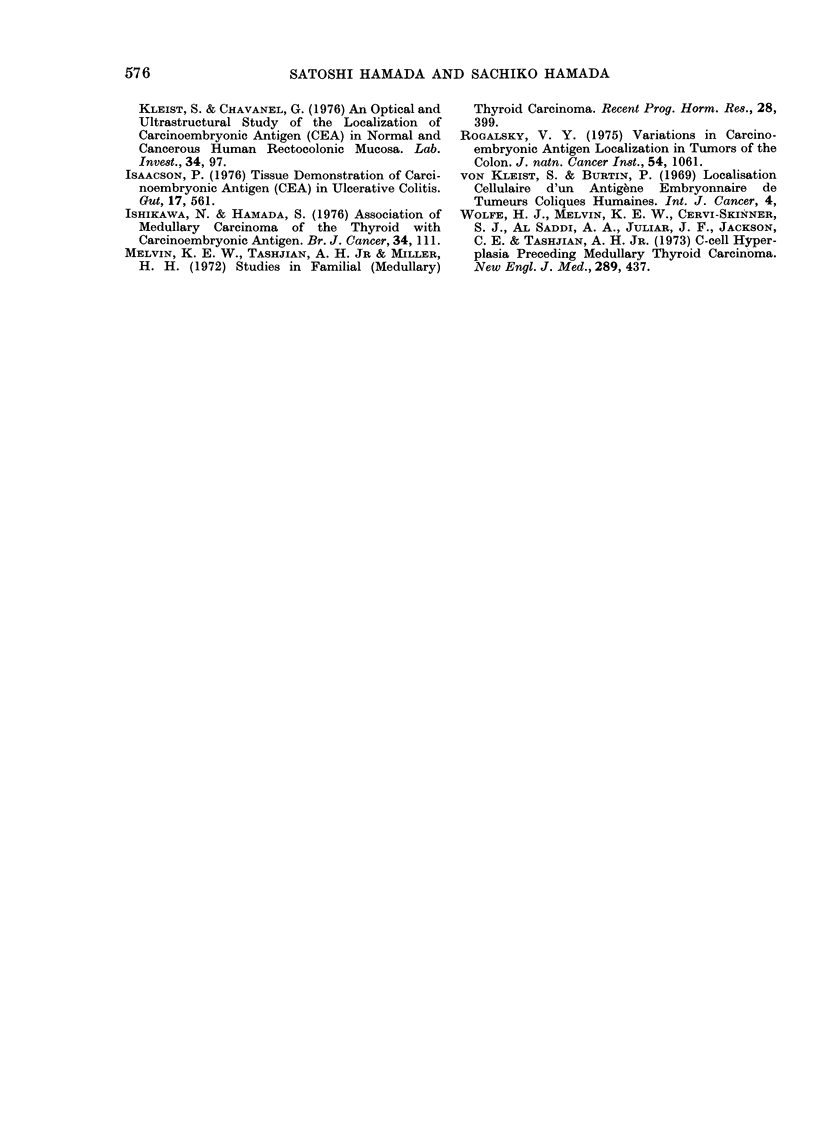# Localization of carcinoembryonic antigen in medullary thyroid carcinoma by immunofluorescent techniques.

**DOI:** 10.1038/bjc.1977.233

**Published:** 1977-11

**Authors:** S. Hamada, S. Hamada

## Abstract

**Images:**


*Br. J. Cancer* (1977) **36**, 572

### LOCALIZATION OF CARCINOEMBRYONIC ANTIGEN IN MEDULLARY THYROID CARCINOMA BY IMMUNOFLUORESCENT TECHNIQUES

SATOSHI HAMADA* AND SACHIKO HAMADA†

*From the Radioisotope Research Center, Kyoto University* and Department of Ophthalmology, Ehime University School of Medicine†*

Received 7 January 1977 Accepted 22 June 1977

**Summary.—Cellular localization of carcinoembryonic antigen (CEA) in medullary thyroid carcinoma was studied in ethanol-fixed, paraffin-embedded specimens using the direct and indirect immunofluorescent techniques. It was demonstrated that CEA was present not only on the surface, but also in the cytoplasm of tumour cells. The immunofluorescence in the cytoplasm differed considerably in intensity from cell to cell. By contrast, no significant fluorescence was demonstrated in tissues of other types of thyroid adenocarcinoma, adenoma, Graves' disease and normal thyroid, with few exceptions. The results obtained indicate that CEA is actively produced by the tumour cells, and is present as a constituent of the cell membrane.**

MEDULLARY carcinoma of the thyroid (MCT), which originates from the parafollicular cell, is distinguished by production of various bioactive substances such as calcitonin (CT), histaminase, etc. (Melvin, Tashjian and Miller, 1972). Quite recently we have demonstrated remarkably high concentrations of carcinoembryonic antigen (CEA) in sera and tumour tissues obtained from MCT, but rarely in other histological types of thyroid carcinomas (Ishikawa and Hamada, 1976). Serum CEA level bore a significant positive correlation with serum CT level, but was not affected by calcium infusion, indicating a secretory mechanism distinct from that of CT (Hamada *et al.*, 1976).

The present study has been performed to localize CEA in MCT employing the direct and indirect immunofluorescent techniques.

### MATERIALS AND METHODS

*Tissue.*—Twelve tissue specimens were obtained at surgery from one patient with medullary carcinoma, 4 with papillary adenocarcinoma, one with papillofollicular adenocarcinoma, one with follicular adenocarcinoma, 3 with follicular adenoma and 2 with Graves' disease. Two normal specimens were obtained from normal thyroid tissue adjacent to the adenoma.

*Immunological reagents.*—Anti-CEA serum was produced by immunization of rabbits with a mixture of purified CEA (Ishikawa and Hamada, 1976) and complete Freund's adjuvant, and was further absorbed with normal pooled serum and normal tissue extracts of colon, lung and liver. The absorbed antiserum thus obtained formed no precipitin line in immunodiffusion against the normal tissue extracts.

Further, $\gamma$-globulin of the absorbed antiserum was conjugated with tetramethylrhodamine by Dr Hiroyuki Ogawa, Department of Internal Medicine II, Kyoto University School of Medicine, and was used for the direct Coons method.

Anti-rabbit IgG antibody conjugated with fluorescein isothiocyanate (FITC) was commercially available from the Fujizoki Company, Tokyo, Japan.

*Indirect and direct immunofluorescent staining.*—Ethanol fixation followed by paraffin-embedding was employed according to the

Reprint requests to: Satoshi Hamada, M.D., Radioisotope Research Center, Kyoto University, Kyoto 606, Japan.

method of Hamashima, Harter and Coons (1964) with a minor modification. Tissue specimens sliced with a thickness of less than 5 mm were fixed in cold 95% ethanol, and dehydrated in successive baths with cold absolute ethanol. The fixed specimens were then embedded in paraffin after xylene treatment.

Sections in thickness of 4 $\mu$m were cut from the tissue blocks, and hydrated in successive baths with xylene and decreasing concentrations of ethanol. After a final wash with 0·01M phosphate-buffered saline (PBS) at pH 7·2, the sections were subjected to immunofluorescent staining and also haematoxylin–eosin staining for structural identification.

In the indirect Coons method, sections were incubated with the absorbed anti-CEA serum at 37 °C for 2 h. After the antiserum was washed off with PBS, they were stained with the FITC-conjugated anti-rabbit IgG $\gamma$-globulin at 37 °C for 2 h. Excessive conjugate was then washed off with large amount of PBS.

In the direct Coons method, sections were stained with the rhodamine-conjugated anti-CEA antibody at room temperature overnight, and then rinsed with large amount of PBS.

*Controls.*—Control staining was always run simultaneously employing normal rabbit serum in place of anti-CEA antiserum. Furthermore, antibodies absorption with purified CEA was performed as a control experiment. Purified CEA was obtained from perchloric-acid extracts of human colonic carcinoma by sequential gel filtration on Sepharose 4B and Sephadex G-200, followed by preparative disc gel electrophoresis (Ishikawa and Hamada, 1976).

### RESULTS

### *Immunofluorescence in medullary carcinoma of the thyroid*

An indirect immunofluorescent technique shows that the medullary carcinoma cells exhibited a strong green fluorescence on the cell surface and also in the cytoplasm (Figs. 1 and 2). The cytoplasmic fluorescence often appeared granular, but the nuclei remained unstained. The immunofluorescence varied considerably from cell to cell.

Larger magnification of the same preparation is shown in Fig. 2. Bright fluorescence was localized along the rim of tumour cells and also within the cytoplasm in some of the cells.

In control staining, using normal rabbit serum in place of the antiserum, no significant fluorescence was seen in the tumour cells. Furthermore, when the anti-CEA serum had been absorbed with a sufficient amount of purified CEA, the fluorescence of the immunofluorescent cells was totally abolished.

Similar findings were obtained by the direct Coons method employing specific anti-CEA antibody labelled with rhodamine. As shown in Figs. 3 and 4, brightly immunofluorescent cells were identified irregularly interspersed in the tumour tissue. Immunofluorescence of these cells was located on the cell surface and also in the cytoplasm, with conspicuous granulation. In other tumour cells, however, the immunofluorescence was much less intense or almost absent.

### *Immunofluorescence in other thyroid tissues*

The tissues obtained from papillary and follicular adenocarcinoma of the thyroid, thyroid adenoma, Graves' disease and normal thyroid were examined under the same conditions. However, no specific fluorescence was noted in any of the tissues examined, except for one case with papillofollicular adenocarcinoma, in which weak fluorescence was observed on the cell surface but not in the cytoplasm by both direct and indirect methods.

### DISCUSSION

The direct and indirect immunofluorescent techniques have shown CEA-specific immunofluorescence in medullary carcinoma (MCT). By contrast, no significant fluorescence was detected in other histological types of carcinomas and benign diseases of the thyroid, except for one case of papillofollicular adenocarcinoma. The results obtained are consistent with the highly specific association of increased

CEA levels with MCT (Ishikawa and Hamada, 1976; Hamada *et al.*, 1976) and, further, provide direct evidence for production of CEA by this tumour.

The cellular localization of CEA in MCT is similar but not identical to that previously reported in gastrointestinal tract tissues. In some cells of MCT the immunofluorescence was seen around the cell surface, but was almost absent from the cytoplasm. The finding is quite similar to those shown in carcinoma or polyp of the colon, in which CEA is located on the luminal cell surface (Gold, Gold and Freedman, 1968; von Kleist and Burtin, 1969; Denk *et al.*, 1972; Burtin *et al.*, 1972, 1973; Bordes, Michiels and Martin, 1973). In other cells, however, bright fluorescence was noted throughout the cytoplasm, exhibiting granulation. Recently, a localized or diffuse distribution of CEA in the cytoplasm has been reported in normal goblet cells and colonic tumour cells (Rogalsky, 1975; Huitric *et al.*, 1976). However, the cytoplasmic immunofluorescence in MCT was different, in both intensity and distribution, from that in these cells.

It has been shown in colonic cells that CEA is secreted from the mucus-secreting cells, and that it is associated with the external coating of epithelial cells (Rogalsky, 1975; Huitric *et al.*, 1976). It appears likely that a similar process may be involved in MCT.

MCT is a calcitonin (CT)-secreting tumour derived from C-cells (Melvin *et al.*, 1972). It is shown by immunofluorescent and immunoenzymatic techniques that this hormone is diffusely distributed within the cytoplasm in the C-cell hyperplasia (Wolfe *et al.*, 1973). It appears, therefore, that CEA differs in its localization from CT.

So far quick-frozen sections, either unfixed or fixed with ethanol, have usually been used for immunofluorescent studies of CEA, although formalin-fixed, paraffin-embedded specimens are usable in the immunoperoxidase techniques (Goldenberg, Sharkey and Primus, 1976; Isaacson, 1976). In the present studies we employed ethanol-fixed, paraffin-embedded tissues, and found them to be more satisfactory than frozen sections. Sections were cut easily in thickness of 4 μm, despite the fragility of the tissue, and were suitable for immunofluorescent staining of CEA.

We wish to thank Prof. Dr Yoshihiro Hamashima, Department of Pathology, Kyoto University School of Medicine, for his invaluable advice and guidance in this study, Dr Phil Gold, Montreal General Hospital, Montreal, Canada, for kindly supplying purified CEA, Dr Hiroyuki Ogawa, Department of Internal Medicine II, Kyoto University School of Medicine, for his labelling of antiserum with rhodamine, and Dr Kanji Kuma, Kuma Hospital, Kobe, for kindly supplying patients' material.

### REFERENCES

BORDES, M., MICHIELS, R. & MARTIN, F. (1973) Detection by Immunofluorescence of Carcinoembryonic Antigen in Colonic Carcinoma, Other Malignant or Benign Tumours, and Noncancerous Tissues. *Digestion*, **9**, 106.

BURTIN, P., MARTIN, E., SABINE, M. C. & VON KLEIST, S. (1972) Immunological Study of Polyps of the Colon. *J. natn. Cancer Inst.*, **48**, 25.

BURTIN, P., VON KLEIST, S., SABINE, M. C. & KING, M. (1973) Immunohistological Localization of Carcinoembryonic Antigen and Nonspecific Cross-reacting Antigen in Gastrointestinal Normal and Tumoral Tissues. *Cancer Res.*, **33**, 3299.

DENK, H., TAPPEINER, G., ECKERSTORFER, R. & HOLZNER, J. H. (1972) Carcinoembryonic Antigen (CEA) in Gastrointestinal and Extragastrointestinal Tumors and its Relationship to Tumorcell Differentiation. *Int. J. Cancer*, **10**, 262.

GOLD, P., GOLD, M. & FREEDMAN, S. O. (1968) Cellular Location of Carcinoembryonic Antigens of the Human Digestive System. *Cancer Res.*, **28**, 1331.

GOLDENBERG, D. M., SHARKEY, R. M. & PRIMUS, F. J. (1976) Carcinoembryonic Antigen in Histopathology: Immunoperoxidase Staining of Conventional Tissue Sections. *J. natn. Cancer Inst.*, **57**, 11.

HAMADA, S., ISHIKAWA, N., YOSHII, M., MORITA, R., FUKUNAGA, M., TORIZUKA, K. & FUKASE, M. (1976) Roles of Circulating Carcinoembryonic Antigen and Calcitonin in Diagnosis of Medullary Thyroid Carcinoma: A Comparative Study. *Endocr. jap.*, **23**, 505.

HAMASHIMA, Y., HARTER, J. G. & COONS, A. H. (1964) The Localization of Albumin and Fibrinogen in Human Liver Cells. *J. Cell Biol.*, **20**, 271.

HUITRIC, E., LAUMONIER, R., BURTIN, P., VON

KLEIST, S. & CHAVANEL, G. (1976) An Optical and Ultrastructural Study of the Localization of Carcinoembryonic Antigen (CEA) in Normal and Cancerous Human Rectocolonic Mucosa. *Lab. Invest.*, **34**, 97.

ISAACSON, P. (1976) Tissue Demonstration of Carcinoembryonic Antigen (CEA) in Ulcerative Colitis. *Gut*, **17**, 561.

ISHIKAWA, N. & HAMADA, S. (1976) Association of Medullary Carcinoma of the Thyroid with Carcinoembryonic Antigen. *Br. J. Cancer*, **34**, 111.

MELVIN, K. E. W., TASHJIAN, A. H. JR & MILLER, H. H. (1972) Studies in Familial (Medullary) Thyroid Carcinoma. *Recent Prog. Horm. Res.*, **28**, 399.

ROGALSKY, V. Y. (1975) Variations in Carcinoembryonic Antigen Localization in Tumors of the Colon. *J. natn. Cancer Inst.*, **54**, 1061.

VON KLEIST, S. & BURTIN, P. (1969) Localisation Cellulaire d'un Antigène Embryonnaire de Tumeurs Coliques Humaines. *Int. J. Cancer*, **4**,

WOLFE, H. J., MELVIN, K. E. W., CERVI-SKINNER, S. J., AL SADDI, A. A., JULIAR, J. F., JACKSON, C. E. & TASHJIAN, A. H. JR. (1973) C-cell Hyperplasia Preceding Medullary Thyroid Carcinoma. *New Engl. J. Med.*, **289**, 437.